# Predictive value of systematic immune-inflammation index combined with Ki-67 index on prognosis of prostate cancer patients after laparoscopic radical prostatectomy

**DOI:** 10.1186/s12894-023-01379-3

**Published:** 2023-12-19

**Authors:** Rongxin Wu, Mingjin Hu, Pei Zhang

**Affiliations:** Department of Urology, Nanjing Lishui People’s Hospital, Zhongda Hospital Lishui Branch, Southeast University, 86 Chongwen Road, Lishui, Nanjing, 211200 China

**Keywords:** Systematic immune-inflammation index, Ki-67 index, Prostate cancer, Laparoscopic radical prostatectomy, Prognosis

## Abstract

**Background:**

Prostate cancer (PCa) presents a wide spectrum. Systemic immune-inflammation index (SII) and Ki-67 index are new biomarkers that can predict prognosis in different types of cancer. We explored the predictive value of their combination on the prognosis of PCa patients after laparoscopic radical prostatectomy (LRP).

**Methods:**

In this retrospective study, 290 patients who underwent LRP at Nanjing Lishui People’s Hospital between January 2016 and February 2021 were enrolled. They were divided into the good prognosis group (N = 235) and poor prognosis group (N = 55) based on the follow-up results. Both the baseline data and postoperative pathological results were collected. The Ki-67 index was determined using immunohistochemical kits, and the patients were allocated to the SII/Ki-67 index high/low expression groups according to the cut-off values to further analyze their relationship with clinical/pathological data of PCa patients. Logistics multivariate regression analysis was utilized to analyze the independent factors affecting post-LRP prognosis of CPa patients. ROC curve was plotted to assess the predictive value for post-LRP prognosis, and Kaplan-Meier curve/Log-rank were used for analysis.

**Results:**

Significant differences were found in PSA/Gleason score/T stage/lymph node metastasis/seminal vesicle invasion/neutrophils/lymphocytes/platelets/preoperative SII/Ki-67 index between the good/poor prognosis groups. Preoperative SII/Ki-67 were related to PSA/lymphocytes/platelets in PCa. Seminal vesicle invasion and preoperative SII + Ki-67 index were independent factors affecting post-LRP prognosis. Preoperative SII + Ki-67 index had a better predictive value than preoperative SII or Ki-67 index alone. Patients with high preoperative SII and Ki-67 index levels had an increased risk of poor prognosis after LRP.

**Conclusion:**

Preoperative SII + Ki-67 index had a better predictive value for poor prognosis after LRP than SII or Ki-67 index alone.

## Introduction

Prostate cancer (PCa) is the most common cancer in men, especially among those over the age of 60, and is the second leading cause of cancer-related deaths worldwide [1]. According to cancer statistics in 2022, the incidence of PCa was about 27%, which dropped rapidly as a result of the prevalence of routine screening [2]. Early detection and accurate diagnosis play a crucial role in the successful management and improved outcomes of PCa, and the diagnostic process typically includes various procedures, such as prostate biopsy, digital rectal examination, prostate-specific antigen (PSA) testing, magnetic resonance imaging, or routine screening. These procedures collectively aid in the identification and evaluation of PCa [3]. Laparoscopic radical prostatectomy (LRP) is a widely employed surgical procedure for treating localized PCa [4, 5]. Despite its effectiveness, there is still a need to identify reliable prognostic factors that can assist in predicting outcomes and guiding postoperative management decisions.

In recent years, the interaction between the immune system and inflammation has emerged as a crucial factor in cancer progression and outcomes [6, 7]. Notably, the systemic immune-inflammation index (SII), defined as neutrophils × platelets/lymphocytes in peripheral blood, is a novel inflammatory marker to reflect the systemic inflammatory microenvironment and reportedly related with poor prognosis for a variety of cancers [8, 9]. An existing study has demonstrated that SII may be used as a significant prognostic marker for PCa patients [10]. On the other hand, the Ki-67 index, a marker of cellular proliferation, has been widely used in cancer research as a measure of tumor aggressiveness [11]. Ki-67 is a nuclear protein expressed in all phases (G1, S, G2 and M) of the cell cycle except the resting G0 phase, and its expression was linked with the proliferation of tumor cells [12]. Several studies have provided supporting evidence of prognostic role for Ki-67 index in PCa after prostatectomy [13, 14]. Considering the potential prognostic significance of both SII and Ki-67 index individually, there is a growing interest in exploring the combined value of these markers in predicting outcomes of PCa patients following LRP. This study aims to investigate the prognostic value of the preoperative SII combined with Ki-67 index in patients undergoing LRP. By evaluating their association with clinical parameters and post-operative outcomes, we seek to determine whether the combined assessment of preoperative SII + Ki-67 index can improve the accuracy of predicting prognosis and improve risk stratification in PCa patients.

## Materials and methods

### Ethics statement

The experiments were authorized by the Academic Ethics Committee of Nanjing Lishui People’s Hospital. All procedures were strictly implemented according to the Declaration of Helsinki. All subjects were fully informed of the the study’s objective and signed the informed consent form prior to sampling.

### Study participants

From January 2016 to February 2021, 366 patients underwent LRP at Nanjing Lishui People’s Hospital were retrospectively selected. However, 45 cases did not meet the inclusion criteria, 15 cases refused to participate in the study, 8 cases dropped out, and 8 cases had incomplete data. Finally, 290 patients were enrolled in the study. The patients were followed up for 2 years. Patients with death or recurrence as the endpoint of follow-up were set as the poor prognosis group (N = 55), and patients with good prognosis during the follow-up as the good prognosis group (N = 235).

### Inclusion criteria

With complete preoperative imaging examination and clinical data; had not received any treatments (such as radiotherapy, chemotherapy, endocrine therapy) that could affect the preoperative SII and Ki-67 index before the operation; confirmed to have PCa based on the postoperative pathological report; underwent LRP.

### Exclusion criteria

With incomplete clinical data or failed follow-up; complicated with other tumors; with self-existing immune diseases and blood diseases; refused to participate in the study or to sign the informed consent form.

### Sample and data collection

The baseline information, including age, self underlying diseases (hypertension, diabetes), PSA value, and blood routine results (neutrophil count, lymphocyte count, platelet count) on the day before surgery after admission, was collected. SII was calculated using the formula: SII = neutrophil count × platelet count/lymphocyte count. The postoperative pathological results, including Gleason score, T stage, lymph node metastasis and seminal vesicle invasion, were recorded.

### Ki-67 index detection

PCa specimens from 290 patients who underwent LRP were collected. The Ki-67 index was determined using a mouse anti-human monoclonal antibody Ki-67 (ZM-0166, Zhongshan Golden Bridge Biological Technology, Beijing, China). The specific operation was carried out in accordance with the instructions of the immunohistochemical kit (Zhongshan Golden Bridge Biological Technology). All specimens were fixed in formaldehyde, embedded in paraffin, sectioned serially using a microtome with a thickness of 4 μm, dewaxed, hydrated, and stained. The results were judged by professional physicians in the pathology department of Nanjing Lishui People’s Hospital. Under the observation of a light microscope, a total of 5 representative high-power fields of view were randomly selected from each section. In each field of view, 1000 cells, either PCa cells or prostate cells, were randomly counted. Brown or tan particles in the nucleus were positive, and the number of positive cells was counted, with their proportion calculated. Its proportion is expressed as the percentage of positive tumor cell number to express Ki-67 index. The Ki-67 index was presented as the percentage of tumor cell positive cells.

### Follow-up

Follow-up was performed every 3 months within the first year after surgery, and once every 6 months in the second year after surgery. Follow-up by telephone/message and outpatient follow-up were used as the follow-up methods for patients. The main method for follow-up was outpatient visits, where they mainly focused on results from tumor marker PSA, chest X-ray, and urinary system CT scans to check for biochemical recurrence. The follow-up endpoint was death or biochemical recurrence.

### Statistical analysis

Statistical analysis and graphing were performed using SPSS 21.0 (IBM Corp, Armonk, NY, USA), GraphPad Prism 8.01 (GraphPad Software, San Diego, CA, USA) and Medcalc (MedCalc Software Ltd, Belgium). The Shapiro-Wilk test was used to test the normal distribution, and the measurement data of normal distribution were expressed as mean ± standard deviation, with the comparisons among groups performed by independent sample *t* test. The measurement data, which followed a non-normal distribution, were represented by the median (minimum, maximum). Group comparisons were conducted using the Mann-Whitney U test. The count data were represented by the number of cases, with the comparisons among groups conducted by Chi-square test. Logistics multivariate regression analysis was utilized to assess independent factors affecting post-LRP prognosis of PCa patients. Receiver operating characteristic (ROC) curve was plotted to analyze the predictive value of preoperative SII/Ki-67 index alone or preoperative SII combined with Ki-67 on the poor prognosis after LRP. According to the distribution comparison of Kaplan-Meier curve and Log-rank test, the poor prognosis curve of PCa patients after LRP was plotted.

## Results

### Clinical baseline characteristics

The enrolled 290 patient were divided into good prognosis group (N = 235) and poor prognosis group (N = 55) according to the follow-up results. The clinical baseline data of the eligible subjects were statistically analyzed. As shown in Table 1, there were no significant differences in age, hypertension, and diabetes between the 2 groups (all *P* > 0.05). However, prominent differences were observed in PSA, Gleason score, T stage, lymph node metastasis, seminal vesicle invasion, neutrophils, lymphocytes, platelets, preoperative SII and Ki-67 index (all *P* < 0.05).

**Table 1:** Clinical baseline characteristics of the enrolled subjects

Parameters	Good prognosis group (N = 235)	Poor prognosis group (N = 55)	*P*
Age (year)	66.46 ± 5.21	67.53 ± 4.61	0.1637
**Hypertension**			
No	165 (70.21%)	39 (70.91%)	0.6694
Yes	70 (29.79%)	16 (29.09%)	
**Diabetes**			
No	190 (80.85%)	45 (81.82%)	0.8692
Yes	45 (19.15%)	10 (18.18%)	
**PSA (ng/mL)**			
< 20	140 (59.57%)	20 (36.36%)	0.0018
≥ 20	95 (40.43%)	35 (63.64%)	
**Gleason score**			
< 8	135 (57.45%)	22 (40.00%)	0.0194
≥ 8	100 (42.55%)	33 (60.00%)	
**T stage**			
T2	109 (46.38%)	16 (29.09%)	0.0197
T3-4	126 (53.62%)	39 (70.91%)	
**Lymph node metastasis**			
No	144 (61.28%)	25 (45.45%)	0.0322
Yes	91 (38.72%)	30 (54.55%)	
**Seminal vesicle invasion**			
No	137 (58.30%)	24 (43.64%)	0.0489
Yes	98 (41.70%)	31 (56.36%)	
Neutrophils (×10^9^/L)	3.31 (1.24,5.21)	3.62 (2.28,5.48)	0.0166
Lymphocytes (×10^9^/L)	1.84 (0.80,3.25)	1.45 (0.82,2.41)	< 0.0001
Platelets (×10^9^/L)	229 (163,355)	284 (207,332)	< 0.0001
Preoperative SII	423.70 (117.10,1197)	692.10 (275.7,1275)	< 0.0001
Ki-67 index	13.84 ± 2.47	18.14 ± 3.38	< 0.0001

Note: Counting data were expressed by number of cases and percentages, and Chi-square test was used for comparisons among groups. The age conformed to the normal distribution, expressed as mean ± standard deviation, and tested by the independent sample *t* test; the neutrophil count, lymphocyte count, platelets, and preoperative SII conformed to the non-normal distribution, expressed as median (minimum, maximum), and tested by Mann-Whitney U test. Ki-67 index conformed to normal distribution was expressed as mean ± standard deviation and tested by independent sample *t* test

### Relationship between preoperative SII and Ki-67 index with clinicopathological data in patients with PCa

To further compare and analyze the relationship between the preoperative SII and Ki-67 index with the clinical data of PCa patients in the 2 groups, patients were divided into SII low/high expression groups and Ki-67 low/high expression groups according to the ROC cut-off values. The data revealed that there were no marked differences in Gleason score, T stage, lymph node metastasis and seminal vesicle invasion between the SII high/low expression groups (all *P* > 0.05), but the proportion of patients with PSA ≥ 20 was higher (all *P* < 0.05), the numbers of neutrophils and platelets were higher, and the number of lymphocytes was lower in the high expression group than the low expression group (all *P* < 0.05) (Table 2). Relative to the Ki-67 low expression group, the Ki-67 high expression group had no significant differences in T stage, seminal vesicle invasion and neutrophil count (all *P* > 0.05), but increased proportions of patients with PSA ≥ 20, Gleason score ≥ 8 and lymph node metastasis (all *P* < 0.05), reduced number of lymphocytes, and increased platelet count (all *P* < 0.05) (Table 2). The aforesaid results displayed that preoperative SII and Ki-67 index were related to PSA, lymphocyte number and platelet level in PCa patients.

**Table 2:** Relationship between preoperative SII/Ki-67 index and clinical data of prostate cancer patients in two groups

Parameters	SII low expression group (N = 145)	SII high expression group (N = 145)	*P*	Ki-67 low expression group (N = 145)	Ki-67 high expression group (N = 145)	*P*
**PSA (ng/mL)**						
< 20	90 (62.07%)	70 (48.28%)	0.0182	89 (61.38%)	71 (48.97%)	0.0336
≥ 20	55 (37.93%)	75 (51.72%)		56 (38.62%)	74 (51.03%)	
**Gleason score**						
< 8	76 (52.41%)	81 (55.86%)	0.5557	88 (60.69%)	69 (47.59%)	0.0251
≥ 8	69 (47.59%)	64 (44.14%)		57 (39.31%)	76 (52.41%)	
**T stage**						
T2	65 (44.83%)	60 (41.38%)	0.5533	62 (42.76%)	63 (43.45%)	0.9056
T3-4	80 (55.17%)	85 (58.62%)		83 (57.24%)	82 (56.55%)	
**Lymph node metastasis**						
No	91 (62.76%)	78 (53.79%)	0.1216	93 (64.14%)	76 (52.41%)	0.0429
Yes	54 (37.24%)	67 (46.21%)		52 (35.86%)	69 (47.59%)	
**Seminal vesicle invasion**						
No	78 (53.79%)	83 (57.24%)	0.5546	83 (57.24%)	78 (53.79%)	0.5546
Yes	67 (46.21%)	62 (42.76%)		62 (42.76%)	67 (46.21%)	
Neutrophils (× 10^9^/L)	2.84 ± 0.75	3.78 ± 0.71	< 0.0001	3.34 ± 0.85	3.28 ± 0.89	0.5097
Lymphocytes (× 10^9^/L)	2.12 (1.25,3.25)	1.48 (0.80,2.33)	< 0.0001	1.86 (0.80,3.25)	1.68 (0.82,3.02)	0.0042
Platelets (× 10^9^/L)	227.50 ± 25.73	251.80 ± 36.22	< 0.0001	229.0 (163,355)	242.0 (167,332)	0.0336

Note: Counting data were expressed by number of cases and percentages, and Chi-square test was used for comparisons among groups. The data conforming to the normal distribution were expressed as mean ± standard deviation, and tested by the independent sample *t* test; the measurement data of non-normal distribution were represented by median (minimum, maximum), and tested by Mann-Whitney U test

### Logistics multivariate regression analysis of the prognosis of PCa patients after LRP

Taking the post-LRP prognosis as the dependent variable (poor prognosis = 1, good prognosis = 0), and PSA, Gleason score, T stage, lymph node metastasis, seminal vesicle invasion, preoperative SII/Ki-67 index with *P* < 0.05 in Table 1 as the independent variables, Logistics multivariate regression analyses were performed. The results elicited that seminal vesicle invasion and preoperative SII/Ki-67 index were the independent factors affecting the prognosis of PCa patients after LRP (Table 3).

**Table 3:** Logistics multivariate regression analysis of the prognosis of PCa patients after LRP

Factor	*P*	OR	95% CI
PSA	0.167	0.526	0.211–1.309
Gleason score	0.258	0.590	0.236–1.472
T stage	0.677	0.820	0.322–2.086
Lymph node metastasis	0.859	0.919	0.361–2.335
Seminal vesicle invasion	0.018	0.315	0.121–0.818
Preoperative SII (× 10^2^)	0.000	2.009	1.594–2.529
Ki-67 index	0.000	1.789	1.475–2.171

### Predictive value of preoperative SII combined with Ki-67 index for poor prognosis after LRP

**Fig. 1:**
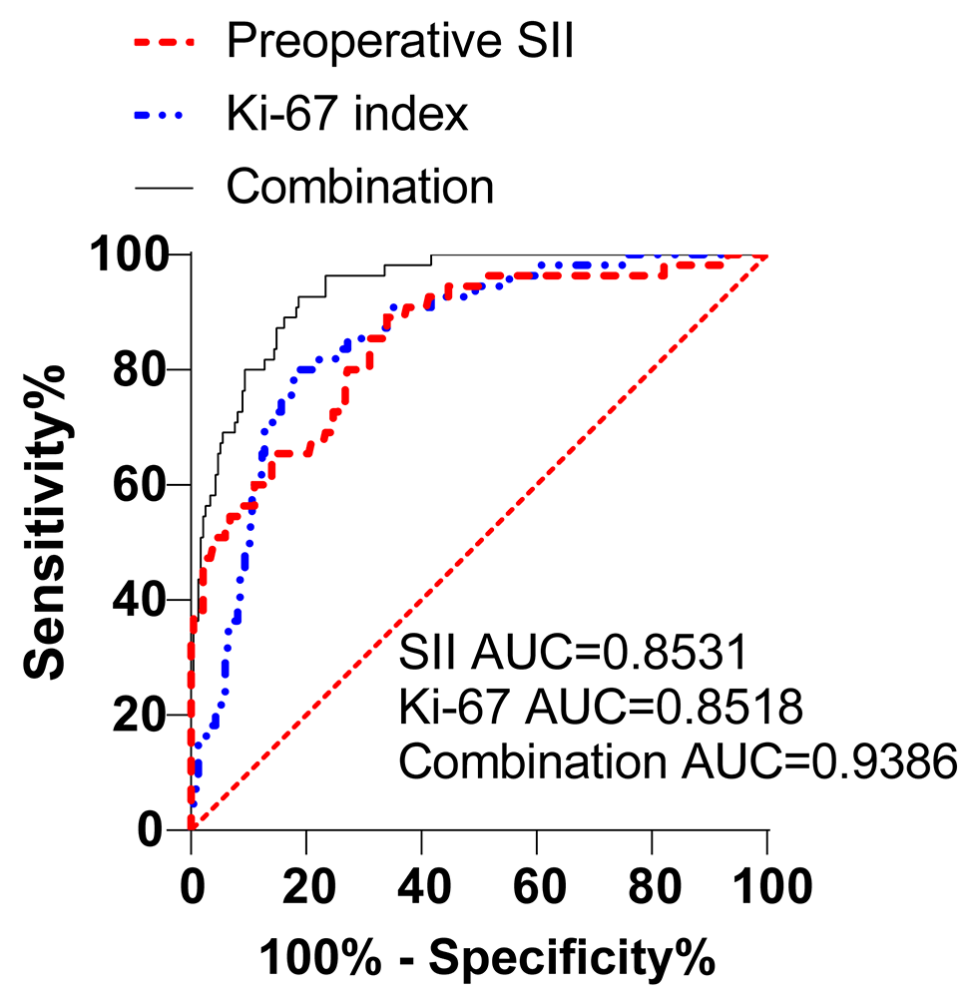
Predictive values of preoperative SII, Ki-67 index and preoperative SII combined with Ki-67 index for poor prognosis after LRP. ROC curve to analyze the predictive values of preoperative SII, Ki-67 index or preoperative SII combined with Ki-67 on the poor prognosis after LRP.

To further evaluate the predictive value of preoperative SII combined with Ki-67 index on the poor prognosis after LRP, the ROC curve analysis was performed with the poor prognosis after LRP as the dependent variable, and the preoperative SII and Ki-67 index as the independent variables, and the results demonstrated that the area under the curve (AUC) of preoperative SII for predicting poor prognosis after LRP was 0.8531, with a sensitivity of 80.00%, and specificity of 82.13%, using a cut-off value of 580.0) (Fig. 1). The AUC of Ki-67 index was 0.8518 (89.09% sensitivity, 65.96% specificity, with the cut-off value of 14.67) (Fig. 1). Furthermore, the AUC of preoperative SII combined with Ki-67 index was 0.9386 (92.73% sensitivity, 81.28% specificity, with the cut-off value of 0.1325 (Fig. 1). Based on the aforementioned findings, it can be summarized that both preoperative SII or Ki-67 index had predictive value for the poor prognosis after LRP, with the combination of preoperative SII and Ki-67 index manifesting a superior predictive value compared to preoperative SII or Ki-67 index alone (Table 4).

**Table 4:** Pairwise comparison of ROC curves

Pairwise comparison of ROC curves	*P*
Preoperative SII vs. Ki-67 index	0.9762
Preoperative SII vs. Preoperative SII + Ki-67 index	0.0002
Ki-67 index vs. Preoperative SII + Ki-67 index	0.0005

### High preoperative SII + Ki-67 index level increased the risk of poor prognosis in PCa patients after LRP

**Fig. 2:**
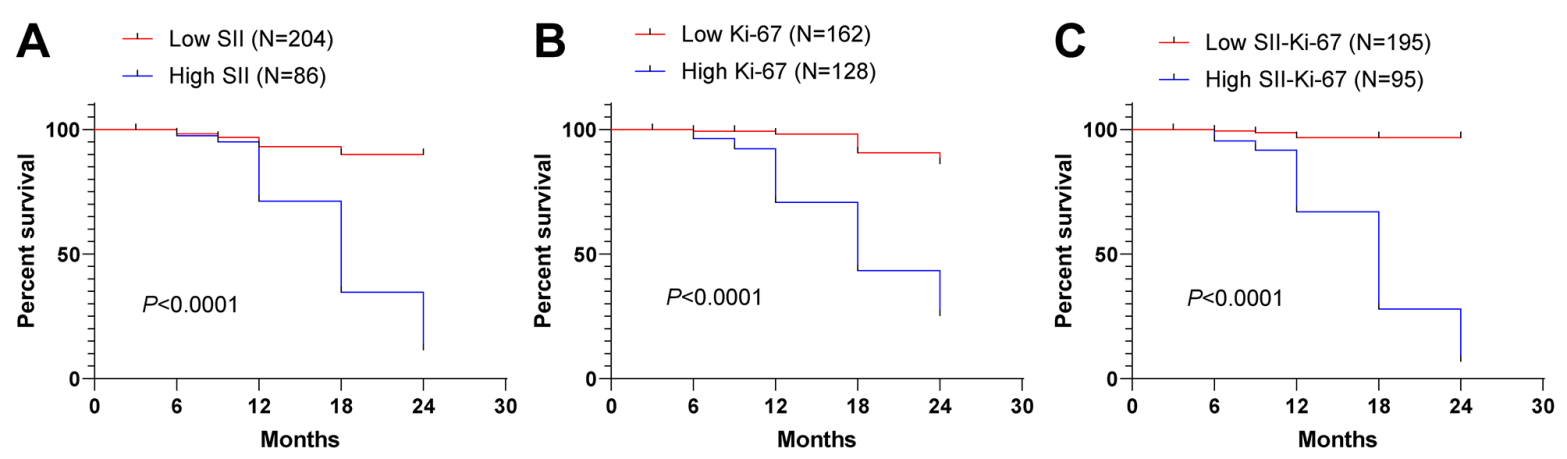
High levels of SII, Ki-67 and preoperative SII combined with Ki-67 index increased the risk of poor prognosis in PCa patients after LRP.

To investigate the correlation between preoperative SII, Ki-67 index and poor prognosis after LRP in patients with PCa, the two-year follow-up prognosis data were analyzed and compared. Compared with the SII-Ki-67, Ki-67, and SII low expression groups, the SII-Ki-67/Ki-67/SII high expression groups exhibited obviously higher rates of poor prognosis (all *P* < 0.0001). Moreover, the poor prognosis rate of the SII-Ki-67 high expression group (53.68%) was found to be higher than that of the SII high expression group (51.16%) and Ki-67 high expression group (37.50%) (Table 5). According to the distribution comparison of Kaplan-Meier curve and Log-rank test, it was observed that the KM curves shifted towards the left in the SII/Ki-67/SII-Ki-67 high expression groups, as compared to their corresponding low expression groups (all *P* < 0.0001) (Fig. 2), indicating that high levels of preoperative SII, Ki-67 index, and preoperative SII combined with Ki-67 index were associated with increased risk of poor prognosis in PCa patients after LRP.

**Table 5:** Prognosis of preoperative SII/Ki-67 index/preoperative SII combined with Ki-67 index high/low expression in two groups

	SII low expression group (N = 204)	SII high expression group (N = 86)	Ki-67 low expression group (N = 162)	Ki-67 high expression group (N = 128)	SII-Ki-67 low expression group (N = 195)	SII-Ki-67 high expression group (N = 95)
Good prognosis	193 (94.61%)	42 (48.84%)	155 (95.68%)	80 (62.50%)	191 (97.95%)	44 (46.32%)
Poor prognosis	11 (5.39%)	44 (51.16%)	7 (4.32%)	48 (37.50%)	4 (2.05%)	51 (53.68%)
*P*	< 0.0001		< 0.0001		< 0.0001	

## Discussion

PCa continues to be the most prevalent diagnosed non-skin malignancy among males, remaining a significant contributor to cancer-related mortality worldwide [15]. Approximately 15% of the patients with PCa are diagnosed with high-risk disease [16]. LRP is a minimally invasive and effective operation to remove the entire prostate gland [4]. The prognosis of PCa patients undergoing LRP often affected by various factors [17]. Accordingly, this present study aimed to investigate the value of preoperative SII combined with Ki-67 index in predicting prognosis of PCa patients following LRP. The findings of this study demonstrated notable correlations between the SII/Ki-67 index and PCa prognosis following LRP, and uncovered that preoperative SII + Ki-67 index exhibited a superior predictive capability for post-LRP poor prognosis compared to either the SII or Ki-67 index alone, which provided valuable insights into the prognostic implications.

Compelling evidence have doucumented that SII can be utilized as an important prognostic indicator in patients with tumors such as small cell lung cancer, renal cell carcinoma, metastatic castrate-resistant prostate cancer, and esophageal squamous cell carcinoma [18–21]. As a marker of cell proliferation, Ki-67 has prognostic value independent from other parameters in human malignancies [22]. However, few studies have been reported on the preoperative SII combined with Ki-67 on the prognosis of tumor patients. The optimal SII cut-off value for glioma grading is calculated by ROC analysis, with the results manifesting that the Ki-67 of patients in the high SII group is remarkably hoisted compared to the low SII group [23]. Elevated SII levels are tightly bound up with Ki-67 ≥ 30% in patients with glioma [24]. Previous studies have proved that both SII or Ki-67 index serve as significant prognostic factors for PCa. Specifically, a high preoperative SII has been found to be related with poor overall survival and progression-free survival [13, 25]. However, the predictive values of SII and Ki-67, as well as their combination, have yet to be fully understood. Initially, we grouped PCa patients according to their prognosis, and uncovered that individuals with a poor post-LRP prognosis exhibited elevated levels of preoperative Ki-67 index and SII. Next, we divided patients into SII and Ki-67 low/high expression groups and discovered for the first time that the SII/Ki-67 high expression group exhibited an increase in neutrophil and platelet counts, accompanied by a decrease in lymphocyte count, suggesting a more pronounced inflammatory response and increased cellular proliferation within the tumor microenvironment, as indicated by the elevated pretreatment SII combined with an elevated Ki-67 index. This combination of immune-inflammatory activation and increased tumor cell proliferation could potentially contribute to tumor aggressiveness and lead to an unfavorable prognosis. Subsequent result revealed this point, i.e., high levels of preoperative SII combined with Ki-67 index increased the risk of poor prognosis in PCa patients after LRP. However, the combination of preoperative SII and Ki-67 index in cancers has been rarely studied. Nevertheless, high SII levels are related to some adverse pathological features, bad overall survival and bad biochemical recurrence-free survival in metastatic-castration resistant PCa patients [26]. An elevated SII indirectly signifies compromised immune function in cancer patients and heightened tumor invasiveness [9]. Additionally, higher Ki-67 staining index has also been revealed to be related to a greater risk of distant metastasis and disease-specific survival in locally advanced PCa after receiving definitive external beam radiotherapy [27]. Meanwhile, Romain Mathieu et al. have demonstrated that Ki-67 positive status is a significant prognostic factor for PCa patients after RP [13]. Besides, a high Ki-67 index suggested aggressive tumor growth as an adverse independent factor for predicting prognosis in primary central nervous system lymphoma [28]. The aforementioned studies unquestionably offered corroborative evidence for our findings. Furthermore, the study identified seminal vesicle invasion and preoperative SII/Ki-67 index as independent factors affecting the prognosis of PCa patients after LRP. We have additionally validated the predictive value of both preoperative SII or Ki-67 index for the poor prognosis following LRP using ROC curves. Our findings unveiled that the combination of preoperative SII and Ki-67 index demonstrates a superior predictive value compared to either preoperative SII or Ki-67 index alone.

In conclusion, preoperative SII combined with Ki-67 index demonstrated a superior predictive value for identifying patients at risk of poor prognosis after LRP. Those mentioned biomarkers, in conjunction with additional clinicopathological factors, have the potential to assist in the process of risk stratification and personalized treatment decision-making for PCa patients. These findings have underscored the multifactorial nature of PCa prognosis and have emphasized the importance of incorporating diverse clinicopathological parameters and molecular markers in the assessment of patients’ outcomes. The inclusion of pre-treatment SII and Ki-67 index in prognostic models could enhance their accuracy and predictive power. It is important to note that this study has some limitations. Firstly, it was conducted retrospectively, potentially introducing inherent biases and limiting the generalizability of the findings. Prospective studies with larger sample sizes and longer follow-up periods are warranted to validate the results. Secondly, the study focused specifically on PCa patients after LRP, and the findings might not be directly applicable to other treatment modalities.

## Data Availability

The data that support the findings of this study are available from the corresponding author upon reasonable request.
